# Models for Predicting Response to Immunotherapy and Prognosis in Patients with Gastric Cancer: DNA Damage Response Genes

**DOI:** 10.1155/2022/4909544

**Published:** 2022-12-19

**Authors:** Rui Dong, Shuran Chen, Fei Lu, Ni Zheng, Guisen Peng, Yan Li, Pan Yang, Hexin Wen, Quanwei Qiu, Yitong Wang, Huazhang Wu, Mulin Liu

**Affiliations:** ^1^Department of Gastrointestinal Surgery, First Affiliated Hospital of Bengbu Medical College, Bengbu, Anhui, China; ^2^School of Life Science, Anhui Province Key Laboratory of Translational Cancer Research, Bengbu Medical College, Bengbu, China; ^3^Department of Gynecologic Oncology, First Affiliated Hospital of Bengbu Medical College, Bengbu, Anhui, China

## Abstract

**Objective:**

DNA damage response (DDR) is a complex system that maintains genetic integrity and the stable replication and transmission of genetic material. m6A modifies DDR-related gene expression and affects the balance of DNA damage response in tumor cells. In this study, a risk model based on m6A-modified DDR-related gene was established to evaluate its role in patients with gastric cancer.

**Methods:**

We downloaded 639 DNA damage response genes from the Gene Set Enrichment Analysis (GSEA) database and constructed risk score models using typed differential genes. We used Kaplan-Meier curves and risk curves to verify the clinical relevance of the model, which was then validated with the univariate and multifactorial Cox analysis, ROC, *C*-index, and nomogram, and finally this model was used to evaluate the correlation of the risk score model with immune microenvironment, microsatellite instability (MSI), tumor mutational burden (TMB), and immune checkpoints.

**Results:**

In this study, 337 samples in The Cancer Genome Atlas (TCGA) database were used as training set to construct a DDR-related gene model, and GSE84437 was used as external data set for verification. We found that the prognosis and immunotherapy effect of gastric cancer patients in the low-risk group were significantly better than those in the high-risk group.

**Conclusion:**

We screened eight DDR-related genes (ZBTB7A, POLQ, CHEK1, NPDC1, RAMP1, AXIN2, SFRP2, and APOD) to establish a risk model, which can predict the prognosis of gastric cancer patients and guide the clinical implementation of immunotherapy.

## 1. Introduction

Gastric cancer ranks fifth in the world's incidence of cancer, and it ranks second in the number of tumor-induced deaths [1]. In fact, 80% of patients with early gastric cancer can achieve a five-year survival rate after treatment [2]; unfortunately, many patients with gastric cancer have no obvious early phenotype and are not found to have metastasized until they are admitted for examination [3]. Even when they undergo surgery, patients with advanced gastric cancer have a low five-year survival rate after surgery [4]. In addition, the insensitivity of gastric cancer to postoperative chemotherapy is even more disabling [5], which may be associated with mismatch repair defects (MMRD) and microsatellite instability (MSI) [6, 7]. Therefore, we need to further explore the underlying mechanisms of gastric carcinogenesis and explore new tumor markers to make better therapeutic decisions.

DNA damage is a key step in carcinogenesis, which is associated with a variety of factors that exist in nature, such as UV light [8] and X-rays. Long-term exposure to carcinogenic environments can damage DNA, cause mutations in oncogenes and cancer suppressor genes, and induce cells to acquire proliferative potential [9]. The DNA damage response is a complex system that includes DNA damage sensors, mediator, and effector proteins [10]. When DNA is damaged, the DNA damage response system blocks the cell cycle by activating cycle check protein sites, inhibiting cell proliferation and recruiting damage repair factors [11]. However, DNA damage response can both repair early tumor mutations and resist tumor killing by chemotherapy and radiotherapy [12]. DNA damage repair defects can also increase the frequency of mutations in the tumor genome [13]. In addition, tumors can overuse DNA damage repair to stall apoptosis [14]. Thus, clinical trials have been conducted using DDR-targeted drugs to treat DDR genetic aberrations in prostate cancer [15] and pancreatic ductal adenocarcinoma [16] or to overcome drug insensitivity of small cell lung cancer to immune checkpoint blockers (ICBs) [17], all of which have shown potential therapeutic value, but the use of DDR inhibitors in gastric cancer remains relatively rare.

DNA methylation is considered to be an important biomarker for cancer diagnosis and prognosis [18]. N6-methyladenosine (m6A) is a highly conserved form of DNA modification in eukaryotic cells [19], which mainly affects the function of genome through epigenetic modification and changing the chromatin structure [20]. A large number of studies have shown that m6A modifications at the DNA level, mRNA level, and protein level are involved in cancer pathogenesis and progression [21] and promote drug resistance in tumor cells [22]. However, the effect of m6A affecting DDR gene expression on the prognosis of gastric cancer patients has not been studied.

Therefore, this study analyzed the transcriptome, mutation, and clinical data of patients. Then, we constructed the risk model of m6A-modified DDR-related genes. After validation of the model validity by GEO dataset, we explored the prognostic ability of the risk model for patients with gastric cancer. We explored the immune cell conditions, MSI, and TMB of patients in the risk model to identify evidence for immunotherapy with the aim of providing new ideas for clinical practice.

## 2. Material and Methods

### 2.1. Data Sources

We downloaded the mutation, transcriptome, and clinical data of gastric cancer patients from TCGA database (https://portal.gdc.cancer.gov/) and Gene Expression Omnibus (GEO) database (https://www.ncbi.nlm.nih.gov/geo/), removed the samples with survival time less than 30 days or unknown, and obtained TCGA set (*n* = 337) and a GEO set (GSE84437, *n* = 433). Finally, 771 samples were enrolled in this study (Table 1).

### 2.2. Screening m6A Gene-Modified Prognosis-Related DNA Damage Response Genes

m6A genes were gained from document [23] and the GSEA database (http://www.gsea-msigdb.org/gsea/index.jsp) GOMF_N6_METHYLADENOSINE_CONTAINING_RNA_BRNA_BINDING, and DNA damage response genes were obtained from the GSEA database REACTOME_DNA_REPAIR and GOBP_DNA_REPAIR. We used “limma” R package to extract the expression matrix of DDR gene and m6A gene. Subsequently, we first matched the expression of DDR genes with their survival status for each patient and then screened the DDR genes with coexpression relationship with m6A genes based on |cor| > 0.1 and FDR < 0.05, and “Psych,” “pheatmap,” and “reshape2” R packages were used to map the Sankey map. The Sankey map showed the DDR genes that have coexpression relationship with m6A genes. We performed the Cox analysis to find prognosis-related DDR genes by referring to the “survival” R package and visualized them by drawing forest plots.

### 2.3. NMF Consensus Clustering

We used the “NMF” and “ConsensusClusterPlus” R packages for unsupervised consensus cluster analysis and divided the patients into two subtypes according to the expression of m6A-modified prognosis-related DDR genes. A good clustering effect should be demonstrated by: (1) a smoothly rising cumulative distribution function curve; (2) the number of samples within a single group should not be too small, and (3) the intragroup correlation should be high and intergroup correlation should be low after clustering. We used the “VennDiagram” R package to screen the differential genes between types based on the filtering range of |log2FC| > 0.1 and FDR < 0.05. The “enrichplot” R package was used to analyze the functions and pathway enrichment areas of the differential genes, and the “GSEABase” R package was used to investigate the differences in immune cell infiltration between the typologies.

### 2.4. Construction of the DDR-Related Gene Risk Model

The 337 gastric cancer patients from TCGA database were used as the original sample. 433 gastric cancer patients from the GEO database were used as the validation sample. The former was used to construct the risk model, and the latter was used as an external dataset to validate the significance of the model in a larger number of patients. We used Lasso regression algorithm to reduce the risk of overfitting based on prognosis-related staging difference genes. The best gene was selected as the construct model using the Cox analysis. The model formula was constructed based on gene expression and coefficients: RiskScore = GENEexp∗Coef, with Coef being the Cox regression coefficient. The risk score for each original and validation sample was calculated based on the model formula. Patients with scores above the median value were defined as high-risk patients. The rest were defined as low-risk patients. The “scatterplot3d” R package was used to locate the patients on a 3D cubic map based on their information. Survival analysis was applied on both groups of patients using the “survminer” R package to determine the value of the risk model.

### 2.5. The Clinical Value of Risk Models

We used the univariate Cox analysis to determine the clinical significance of the model for patients, and then, the multifactorial Cox analysis was used to remove the interference of other clinical traits to determine the ability of the risk model to predict patients' survival over the last 5 years without relying on their clinical traits. Then, we combined some clinical manifestations (gender, stage, T stage, N stage, grade, and age) and risk scores of gastric cancer patients to draw a nomogram and visualize it with the “regplot” and “rms” R packages. And calibration curves were used to illustrate the agreement between the actual results and the model-predicted results. We used “survminer” R package-plotted K-M curves to find whether the constructed risk model was applicable to patients of different clinical subgroups.

### 2.6. Correlation of Risk Model and TME

The tumor microenvironment (TME) is essential for tumor cells to survive and exert their malignant phenotype. It controls cancer cell invasion, migration, and drug sensitivity [24]. Study shows that altering TME helps improve treatment effectiveness in cancer cells [25]. In this study, we used the ESTIMATE algorithm to calculate the stromal cell score and immune cell score for each sample and scored them together. The CIBERSORT algorithm was used to calculate the immune cell subpopulation content in gastric cancer samples. Correlation scatter plots were drawn using the “ggplot2” and “tidyverse” R packages to show the immune cell subpopulation content of each risk group. We used ssGSEA to compare the differences in the content of each type of immune cells in the two risk groups.

### 2.7. Correlations of Risk Models with TMB, MSI, and Drug Sensitivity

Tumor mutational burden affects the immune response of tumor cells [26]. In order to assess the frequency of mutations in gastric cancer samples between the two risk groups, the number of mutated genes in gastric cancer patients was counted together and the tumor mutation load (TMB) score was calculated for each patient using the “maftools” R package. We selected the top 20 genes with mutation frequency and plotted the waterfall map of their mutation numbers to analyze the correlation between the risk model scores and the tumor mutation burden scores. We counted the number of mutations in patients and used this as a basis to classify patients into high- and low-TMB groups and compared these two groups as survival status. R packages such as “ggpubr” and “plyr” were used to draw the status of microsatellite instability and immune escape under different risk scores. The “pRRophetic” R package was used to calculate the concentration of multiple antitumor drugs that inhibited half of the tumor growth (IC_50_) to investigate the difference in drug sensitivity between the two groups of patients.

### 2.8. Procedures and Statistical Analysis

All script runners were performed using R version 4.1.2. Statistical significance was set at *p* < 0.05.

## 3. Results

### 3.1. Screening m6A-Modified Prognosis-Related DDR Genes

**Figure 1:**
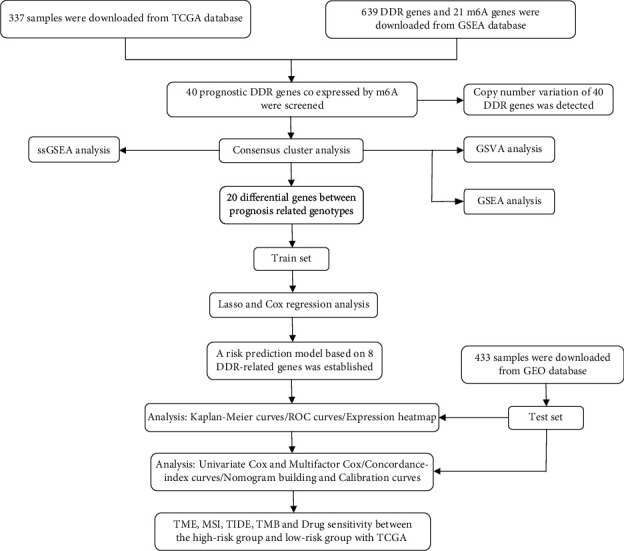
The flowchart describing the experimental design.

**Figure 2:**
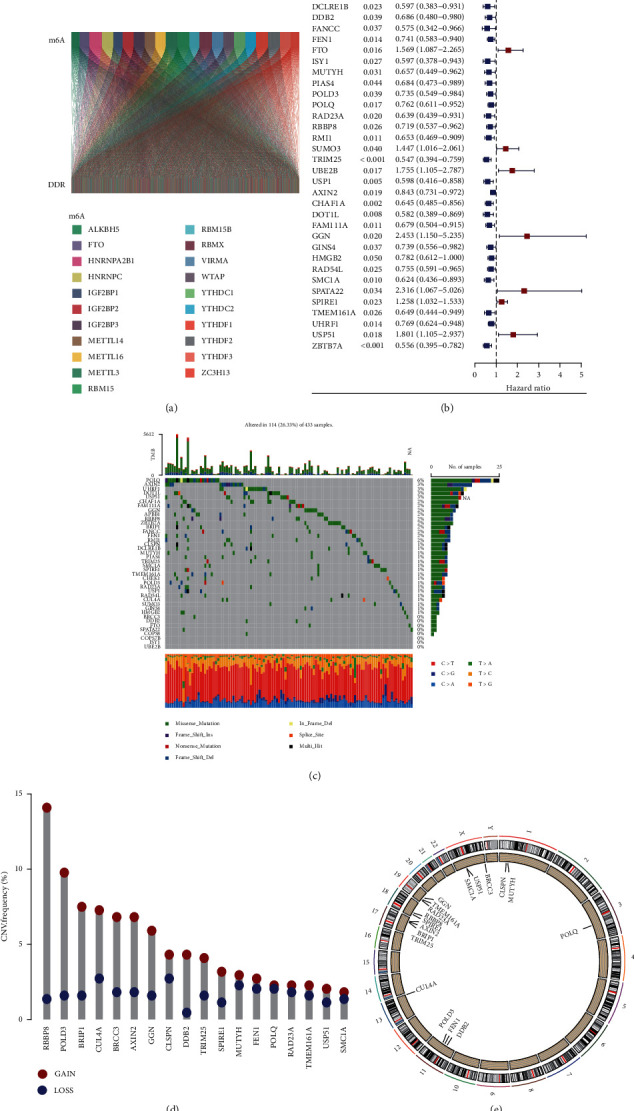
Mutation frequency and copy number variation of m6A-modified prognosis-related DDR genes in patients with gastric cancer. (a) Sankey diagram of m6A genes and DDR genes. (b) Univariate Cox regression prognostic model of DDR genes in patients with gastric cancer. (c) Mutation frequency of 40 prognosis-related DDR genes. (d) Frequency of increase, loss, and noncopy number variation of DDR genes. (e) Location of copy number variation of DDR genes on 23 chromosomes.

The m6A genes have been reported to direct DNA repair factors to DNA double-strand break sites, while the DNA damage response maintains stability of genome, which contributes to cancer progression [27]. To explore the potential biomarkers, we intend to construct a risk model using m6A-modified DDR genes. We first determined the technical route for building the risk model (Figure 1). We searched TCGA database for gastric cancer patients' transcriptome data, collected 639 DDR genes and 21 m6A genes from the GSEA database, and found the m6A-modified DDR genes with *p* value < 0.05 and |cor| > 0.1 (Figure 2(a)). Subsequently, we used the univariate Cox analysis to evaluate the correlations of DDR gene expression with prognosis in gastric cancer samples and screened 40 DDR genes with prognostic characteristics (Figure 2(b)). A summary of the mutation rates of these 40 prognosis-related DDR genes revealed that most DDR genes were mutated, with POLQ having the highest mutation frequency (6%), while 8 DDR genes did not have any mutations (Figure 2(c)). The results of somatic copy number showed that there was a general copy number variation (CNV), and 18 prognosis-related DDR genes increase (Figure 2(d)).  Figure 2(e) shows the location of copy number variation of DDR gene on their respective chromosomes. In conclusion, we screened 40 m6A-modified prognosis-related DDR genes for subsequent analysis.

### 3.2. Identification of m6A-Modified DDR Gene Subtypes in Gastric Cancer

**Figure 3:**
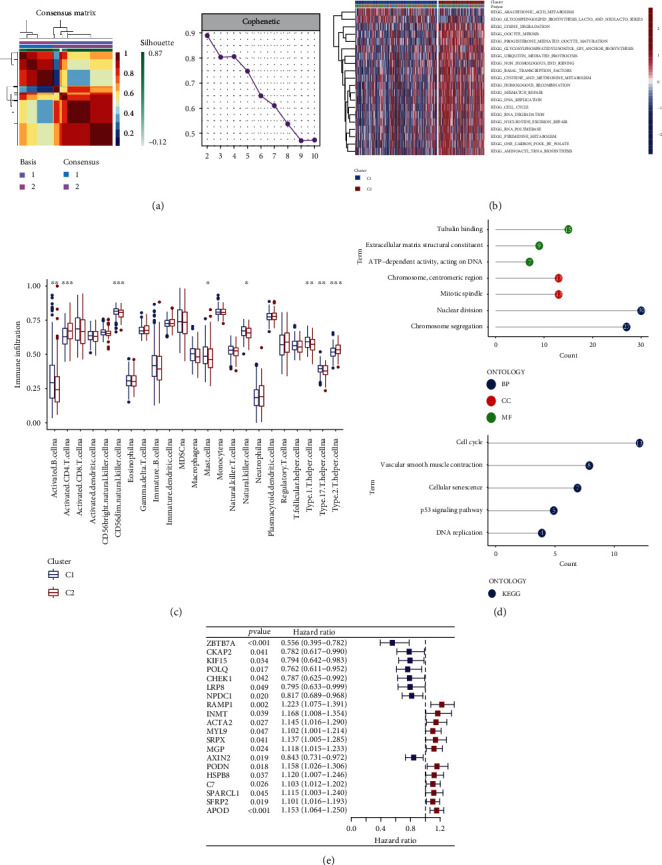
Consensus cluster analysis of m6A-modified prognosis-related DDR genes. (a) Define the consensus matrix of two subtypes (*k* = 2). (b) GSVA enrichment analysis of two subtypes. (c) Immune cell abundance difference between two subtypes. (d) GO and KEGG enrichment analyses of DDR-related differential genes. (e) Univariate Cox regression prognosis model of DDR-related differential genes.

To expand the clarification of the correlated expression of cognitive m6A-modified prognosis-related DDR genes in gastric cancer patients, we attempted to classify patients according to the expression of these 40 DDR genes with prognostic features in patients using the NMF classification. Comparing the results of the full typing, we determined that *k* = 2 most clearly differentiated the patients, and the number of patients in each group met the requirements for subsequent analysis; there were 216 patients in the C1 subtype and 121 patients in the C2 subtype. We found that genes in group C1 were enriched in pathways such as drug metabolism, and genes in group C2 were enriched in pathways such as cell cycle and DNA repair by GSEA of typed differential genes (Figure 3(a), Supplementary Figure 1). Gene Set Variation Analysis (GSVA) of different subtypes showed that subtype C1 was mainly enriched in nutrient metabolic pathways, such as arachidonic acid metabolic pathway and glycosphingolipid biosynthesis lacto; subtype C2 was mainly enriched in DNA damage response pathways, such as mismatch repair and homologous recombination and nucleotide excision repair (Figure 3(b)). We used the ssGSEA algorithm to enrich for immune-related genes and used this to calculate the different abundance of immune cells in the two groups of patients (Figure 3(c)). We compared the gene expression between C1 and C2 subtypes, censored the genes with |logFC| ≤ 1 and *p* value ≥ 0.05, and finally screened 178 differentially expressed genes. We then performed Gene Ontology (GO) and Kyoto Encyclopedia of Genes and Genomes (KEGG) enrichment analyses on these genes, and the results showed that the differential genes were mainly involved in nuclear division and chromosome segregation (Figure 3(d)). These differential genes were combined with patient survival information to screen for 20 prognosis-related differential genes (Figure 3(e)). In conclusion, not only m6A-modified DDR genes but also differential genes (DEGs) after typing are related to cell cycle and participate in cell replication and proliferation.

### 3.3. The DDR-Related Gene Risk Model

To construct a model for DDR-related genes and to investigate its efficiency in assessing the prognosis of gastric cancer patients, we used samples from TCGA database. Based on transcriptomic data and clinical data in TCGA database samples, these datasets were eliminated by applying the “Combat” algorithm. Lasso regression analysis and the multivariate Cox analysis were performed on the above 20 prognosis-related typing difference genes, and 8 best candidate genes, including 3 potential risk genes and 5 potential protective genes, were screened for risk model construction.

RiskScore = (−0.5057∗expression of ZBTB7A) + (−0.0482∗expression of POLQ) + (−0.1283∗expression of CHEK1) + (−0.1406∗expression of NPDC1) + (0.0825∗expression of RAMP1) + (−0.1175∗expression of AXIN2) + (0.0205∗expression of SFRP2) + (0.0357∗expression of APOD).

**Figure 4:**
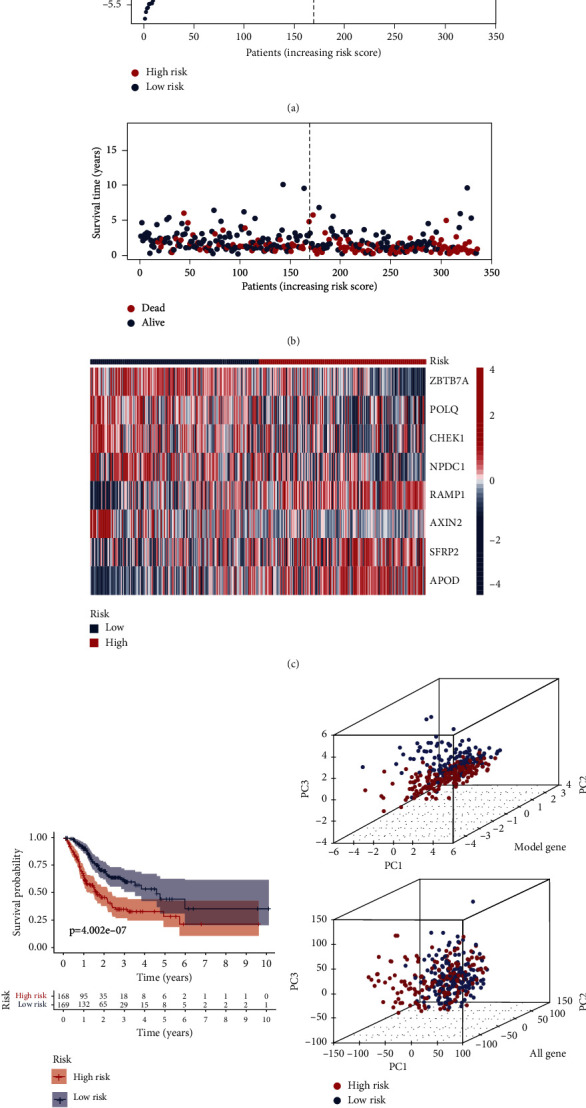
Construction of prognostic models in the TCGA original sample. (a, b) Risk score curves and scatter plots of mortality risk for DDR-related genes. (c) Heat map for visualization of gene expression data for the eight risk model genes. (d) K-M curves of the overall survival of the two groups of patients. (e) Three-dimensional cube plot after principal component analysis based on the transcriptome information of patient risk model genes (top) and all genes (bottom).

We categorized patients with risk scores above the median value into the high-risk group (*n* = 168) and the rest into the low-risk group (*n* = 169). The risk curve results showed that the higher the risk score of the patients, the higher the risk of death (Figures 4(a) and 4(b)).  Figure 4(c) shows the difference in the expression of genes constructing the risk model in the high- and low-risk groups in which ZBTB7A, POLQ, CHEK1, NPDC1, and AXIN2 had lower expression in the high-risk group, suggesting that they are potential protective genes in gastric cancer; RAMP1, SFRP2, and APOD had lower expression in the low-risk group, suggesting that they are potential risk genes in gastric cancer. We plotted K-M curves based on the survival time and survival status of patients, and the statistical results showed that patients in the low-risk group had a significantly better prognosis and longer survival time (Figure 4(d)). We used principal component analysis (PCA) to replace the original variables and display them on a three-dimensional cube plot; we found that the risk model gene transcriptome data were more discriminatory when used to distinguish the space in which the two groups of patients were located (Figure 4(e)). Subsequently, we analyzed the prognosis of gastric cancer patients using other investigators' models genetically and compared them with the model of the present study [28–32], and the results showed that our model distinguished patients in the high- and low-risk groups more significantly and was more sensitive (Supplementary Figure 2). In summary, this study constructed a risk model based on eight DDR-related genes and validated their clinical significance.

### 3.4. Validation of DDR-Related Gene Prognostic Model in GEO Database

**Figure 5:**
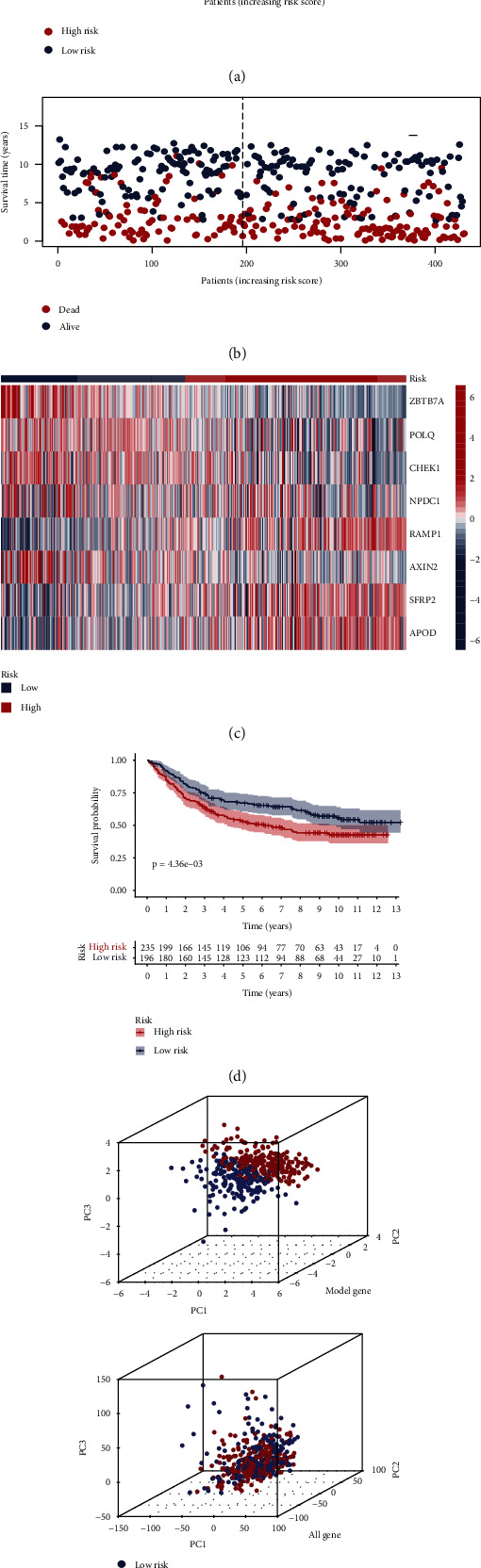
Construction of prognostic models in the GEO validation sample. (a, b) Risk score curves and scatter plots of mortality risk for DDR-related genes. (c) Heat map for visualization of gene expression data for the eight risk model genes. (d) K-M curves of the overall survival of the two groups of patients. (e) Three-dimensional cube plot after principal component analysis based on the transcriptome information of patient risk model genes (top) and all genes (bottom).

To further access the predictive role of the risk model constructed in this study in patients with gastric cancer, we selected GSE84437 as the validation sample and used the same formula as the original sample to calculate risk scores for all samples. We also classified patients with risk scores above the median value into the high-risk group (*n* = 236) and the remaining patients into the low-risk group (*n* = 197). The risk curve results showed that the higher the patient's risk score, the greater the risk of death (Figures 5(a) and 5(b)). We transformed the expression of the eight risk model genes in the validation sample into chroma bonds and plotted heat maps to visualize them, and the heat map results were consistent with the original samples (Figure 5(c)). We then plotted K-M curves based on the survival data of patients in the validation sample, and the risk of death was similarly higher in the high-risk group (Figure 5(d)). We used principal component analysis to downscale the patient information and display it in a three-dimensional cube plot, and Figure 5(e) shows that the difference between the two groups of patient information is more clearly distinguished using the risk model gene transcriptome data. In summary, we validated the validity of the risk model in an external dataset.

### 3.5. Assessment of Risk Genes

**Figure 6:**
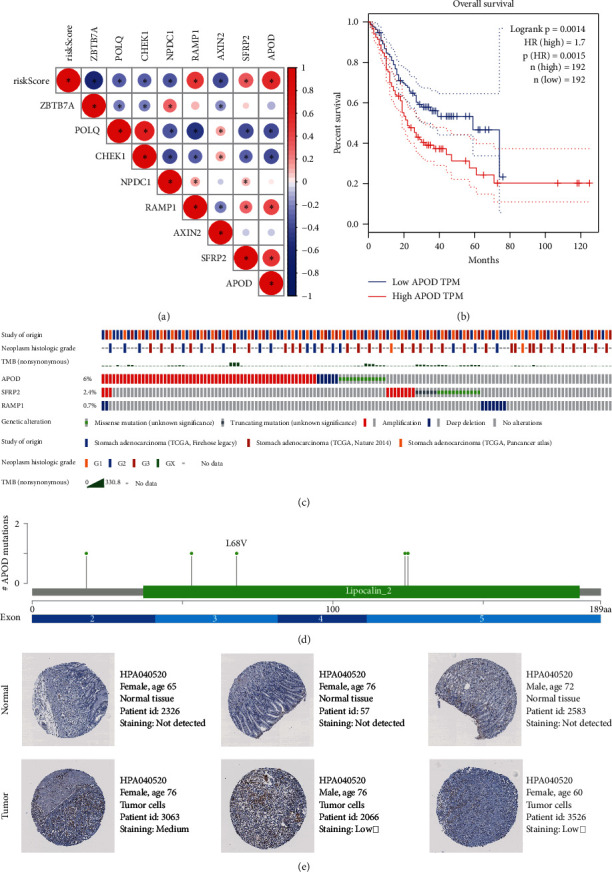
Evaluation of risk model genes. (a) Correlation of risk model genes with risk scores. (b) Overall survival curves of high APOD expression group and low APOD expression group. (c) Mutation rates of three potentially high-risk genes. (d) Mutation loci of APOD. (e) Immunohistochemical results of APOD antigen in normal and tumor tissues.

We plotted the correlation between the risk model genes and the risk scores (Figure 6(a)); APOD, SFRP2, and RAMP1 were significantly positively correlated with risk scores, with APOD having the strongest correlation with risk scores and significantly affecting the prognosis of gastric cancer patients (Figure 6(b)), with patient information from the GEPIA website (http://gepia.cancer-pku.cn/index.html). We searched the cBioPortal database for mutation data of three potential risk genes (Figure 6(c)), in which APOD had the highest mutation rate (6%), followed by SFRP2 (2.4%) and RAMP1 (0.7%), and Figure 6(d) shows the mutation loci of APOD. In addition, we analyzed APOD protein expression by the Human Protein Atlas (https://www.proteinatlas.org/) to find differences in APOD protein expression, and immunohistochemical maps showed that APOD was highly expressed in gastric cancer tissues (Figure 6(d)). In conclusion, APOD is the potentially high-risk gene with the highest mutation rate, and its high expression may promote the progression of gastric cancer.

### 3.6. Clinical Correlation and Prognostic Values of the Prognostic Model

**Figure 7:**
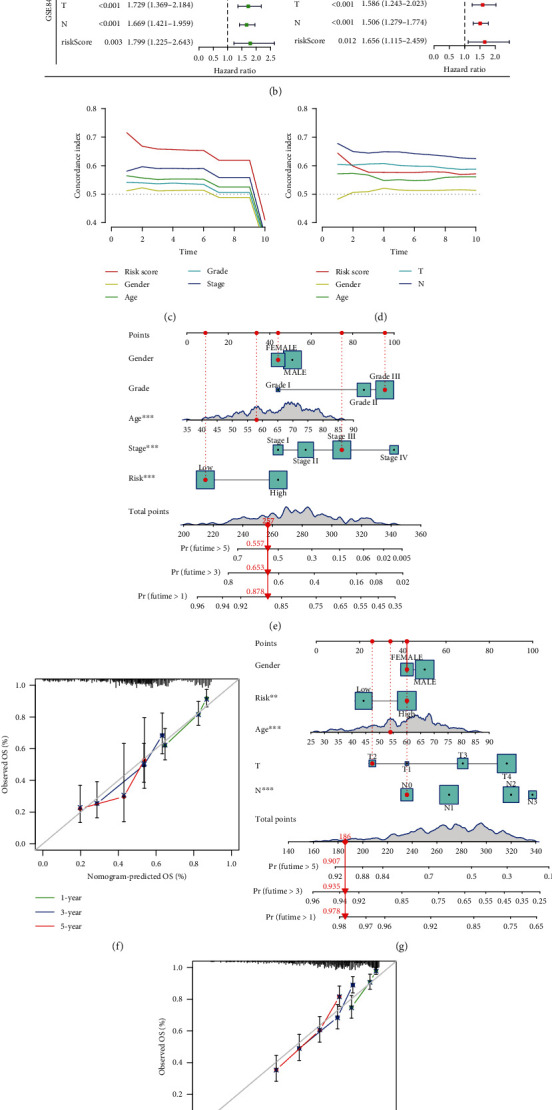
clinical relevance of risk models. (a) Univariate (left) and multivariate (right) Cox regression analysis of the original TCGA samples. (b) Univariate (left) and multivariate (right) Cox regression analysis of the Validation GEO Samples. (c, d) *C*-index charts of risk scores and clinical relevance. (e, f) Nomogram and calibration plots for predicting 5-year survival of gastric cancer patients in the original samples. (g, h) Nomogram and calibration plots for predicting 5-year survival of gastric cancer patients in the validation samples.

To assess the ability of this risk model and to predict survival independent of the patient's clinical traits, this study applied the univariate and multifactorial Cox analyses of age, gender, grade, stage, and risk scores in the TCGA cohort. Stage and DDR-related genetic risk scores have independent prognostic power (*p* < 0.001, HR = 1.585 (95% CI, 1.272–1.976) and *p* < 0.001, HR = 4.665 (95% CI, 2.786–7.811); Figure 7(a)). Univariate and multifactorial Cox analyses of age, gender, T stage, N stage, and risk scores in the GEO cohort showed that DDR-related genetic risk scores also had independent prognostic value (*p* = 0.012, HR = 1.656 (95% CI, 1.115–2.459); Figure 7(b)). Figures 7(c) and 7(d) showed that the *C*-index curve of the risk scores has a high predictive sensitivity over time. We then scored TCGA cohort of gastric cancer patients on the basis of regression coefficients and obtained individual scores for each variable, summing the total scores to calculate the probability of 5-year survival (Figure 7(e)). The nomogram calibration curve shows the deviation from the ideal model (Figure 7(f)). This was also verified in the nomogram and calibration plots of the GEO cohort patients (Figures 7(g) and 7(h)). In addition, we analyzed the applicability of the prognostic model to patients in any of the clinical states by grouping them according to their clinical status, and the K-M curves showed that the prognostic model responded to the survival status of patients with different clinical traits (Supplementary Figure 3A). We also identified prognosis-related DDR difference genes between the two groups (Supplementary Figure 3B). In conclusion, our prognostic model constructed has strong clinical relevance and prognostic values, and its predictive value in immunotherapy can be further explored.

### 3.7. Analysis of the Immune Microenvironment

**Figure 8:**
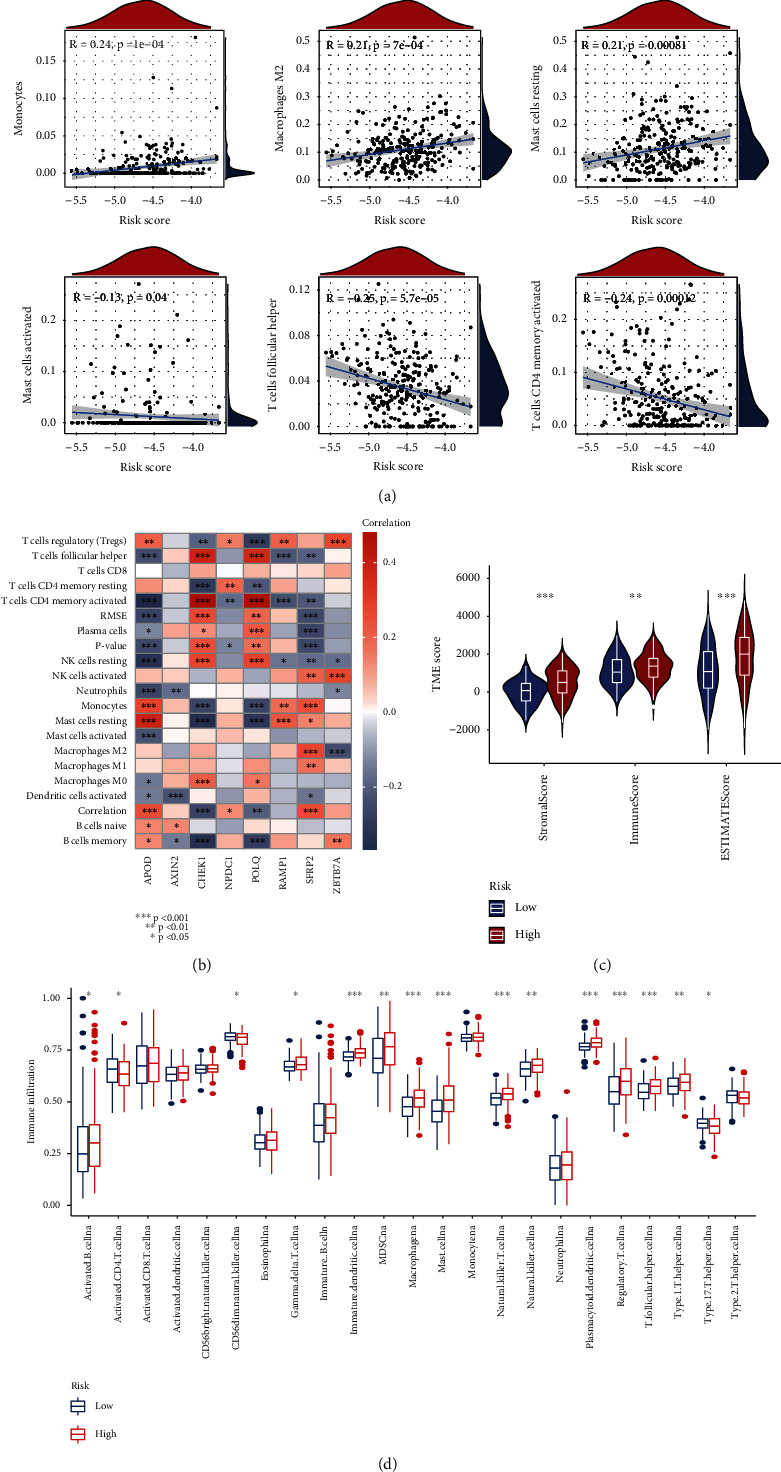
Immune cell infiltration in gastric cancer samples. (a) Spearman's test for correlation of DDR-related gene risk scores with immune cell abundance. (b) Correlation of immune cell abundance and DDR-related model genes. (c) Relationships between eight DDR-related risk scores and both immune and stromal scores. (d) Relationships between eight DDR-related risk scores and immune cell infiltration.

Proliferation, differentiation, and immune efficacy of innate and adaptive immune cells are affected by the DNA damage response [33]. For this purpose, we used the CIBERSORT algorithm to calculate the abundance of immune cell infiltration in each sample and made a scatter plot of its risk score with DDR-related genes. After correlation by Spearman's test, we found that the risk score was positively correlated with monocytes, macrophages M2, mast cell resting, negatively correlated with mast cell activated, T-cell follicular helper, and T-cell CD4 memory activated (Figure 8(a)). In addition, the eight model genes of this study had interaction with the majority of immune cells (Figure 8(b)). We used the ESTIMATE algorithm to estimate stromal cell proportions and immune cell proportions based on transcriptome expression data, which were summarized as a TME scoring file, and then compared the differences in scores between the two groups of patients (Figure 8(c)). In addition, we also used ssGSEA to estimate the abundance of immune cell infiltration and found that there were significant differences between the two groups for 15 immune cells (Figure 8(d)). In conclusion, our model can estimate the content of immune cells and we found that in DDR-related gene models, tumors from different groups of gastric cancer patients had different TME.

### 3.8. Analysis of the MSI, TMB, and Drug Susceptibility

**Figure 9:**
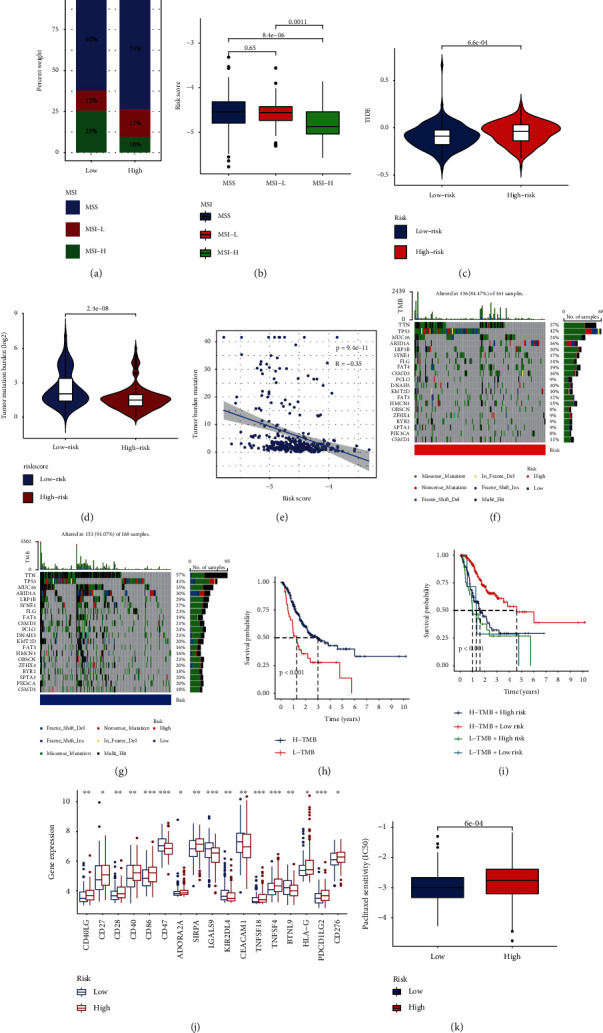
A comprehensive analysis of the response of two groups of gastric cancer patients receiving immunotherapy. (a, b) Association of DDR-related genetic risk scores with MSI. (c) Differences in TIDE scores between the two groups. (d, e) Association of DDR-related genetic risk scores with TMB. (f, g) Waterfall plot of the top 20 genes in somatic mutation frequency in both groups. (h, i) Kaplan-Meier analysis of combined TMB and risk scores in patients with gastric cancer. (j) The relationship between the two risk groups of gastric cancer patients and immune checkpoint. (k) The relationship between the two risk groups of patients and drug sensitivity.

It was reported that modulating DNA damage response will affect the drug resistance of tumor patients to immunotherapy [34]. First, we counted the number of patients in each type of microsatellite instability status in both groups and plotted the percentage histogram. We then analyzed the correlation between the risk score and the number of people in each category; the higher the risk score, the higher the number of people in MSI-L and MSS status (Figures 9(a) and 9(b)). TIDE score is a novel prognostic assessment scheme for immune checkpoint suppression therapy, and our findings showed that patients with high-risk scores have higher TIDE scores (Figure 9(c)), suggesting that they have poorer outcomes with immunotherapy. Immunotherapy is more effective in patients with high TMB because of its ability to provide more neoantigens [26]. We counted each gene mutation in each gastric cancer patient, which was summarized in a table of gene mutation counts, as a way to analyze the TMB differences between the two groups, and we found that the TMB was lower in the high-risk group (Figures 9(d) and 9(e)), which suggests that the high-risk group is not effective in immunotherapy again. We then further counted the mutation frequencies of patients in both groups on each gene. The top 5 genes in mutation frequency were all TTN, TP53, MUC16, ARID1A, and LRP1B, but the mutation frequencies of TTN, MUC16, and ARID1A were significantly lower in the high-risk group patients (Figures 9(f) and 9(g)). In addition, assessing the effect of TMB on the prognosis of gastric cancer patients alone, the K-M curve showed that patients with low TMB had a poorer prognosis (Figure 9(h)), and prognostic analysis combined with risk score showed that patients with low risk score combined with high TMB had the best prognosis (Figure 9(i)). We also focused on the oncogenic effect of using immune checkpoint inhibitors in this model.  Figure 9(j) shows that a total of 17 immune checkpoint genes were significantly different between the two risk groups. Next, we also tried to suggest chemotherapeutic agents for the treatment of gastric cancer based on the expression of genes affecting drug sensitivity in both groups, and we found that patients with low risk scores had lower semi-inhibitory concentration values (IC_50_) for paclitaxel (Figure 9(k)). In summary, patients in the high-risk group had poorer immunotherapy outcomes and poorer prognosis than patients in the low-risk group.

## 4. Discussion

Stomach cancer is a highly prevalent tumor, with more than 1 million new cases diagnosed each year, and the second highest number of deaths from all tumors worldwide [35], and it causes great damage to human health. The development of gastric cancer is related to some uncontrollable factors, such as gender, age, ethnic, and genetics and also controllable factors, such as HP, alcohol consumption, and nitrates. Early signs of gastric cancer patients were without obvious discomfort, and patients often complain of epigastric discomfort, abdominal pain, difficulty in swallowing, vomiting blood, and black stool when they are admitted to the hospital, and at this time, they are mostly in advanced stage, which brings great challenges to the treatment of gastric cancer. Multidisciplinary combination therapies such as surgery, radiotherapy, systemic chemotherapy, immunotherapy, and targeted therapy have opened up more directions for cancer treatment, especially in recent years, immunotherapies targeting immune checkpoints, such as those targeting the PD-1/PD-L1 axis, offer new hope for cancer treatment [36]. Unfortunately, the benefits for gastric cancer patients are very limited [37]. Therefore, there is an urgent need to understand the pathogenesis of gastric cancer and to find effective treatment directions.

In recent years, the role of DNA damage response in cancer has been included in the topic by medical researchers. Such response includes recognition of DNA damage sites, stalling or collapsing replication forks, blocking the cell cycle, and inducing apoptosis [38]. Early in tumorigenesis, the DNA damage response pathway recognizes DNA damage sites, arrests the cell cycle and attempts repair, and induces apoptosis if DNA damage exceeds the repair threshold, but when DDR-related genes are mutated, it can become an accomplice to cancer, saving cancer cells from damage caused by radiation and chemotherapy, allowing cancer cells that should be apoptotic to gain the ability to proliferate again and prolonging the survival of cancer cells [39]. The above analysis suggests that we can identify new biomarkers from DDR-related genes.

There are many researchers looking for potential therapeutic targets by constructing prognostic models, for example, Song et al. studied pyroptosis-related genes and applied them to prognosis of colorectal cancer patients [40]. Xu et al. explored the m6A-related lncRNA and applied them to predict prognosis of lung cancer patients [23]. However, no investigator has predicted the survival prognosis of gastric cancer patients according to the DDR genes. We analyzed the transcription data of DDR genes and survival data of gastric cancer samples together and used Lasso and the Cox regression analysis to mine eight core genes for constructing the model. These DDR-related genes have been studied and found to affect tumor biological behavior in a variety of cancer types, of which we are particularly interested in the risk gene with the highest mutation rate, APOD, whose expression product is tumor apolipoprotein D. Upregulation of APOD expression has been reported to promote the progression of breast cancer [41] and prostate cancer [42], and it may be an important risk gene for gastric cancer. After constructing the model, we divided the patients 1 : 1 into two groups of high and low risk. Analyzing the survival information from both the original and validation samples, we plotted survival curves showing poor prognosis for patients in the high-risk group, and the ROC curves validated the sensitivity of the K-M analysis. Multifactor Cox analysis of risk scores with *p* values < 0.01 indicated that our risk scoring approach could infer the risk of death without relying on patients' clinical performance. The *C*-index curves illustrated that the model outperformed the commonly used clinical grading criteria in predicting the sensitivity of survival of gastric cancer patients. We also converted each clinical index of patients to a single variable and aggregated and plotted nomograms and their calibration curves to provide a new method for assessing 5-year survival of gastric cancer patients.

DNA double-strand breaks (DSBs) activating kinases such as ATM, ATR, and DNA-PKcs-induced DNA damage responses are an important component of immune cell development during antigen signaling assembly [28]. Therefore, this study further investigated the role of this risk model in guiding immunotherapy in gastric cancer patients. Many studies have shown that the tumor microenvironment is a key factor affecting tumor immune escape [43] and alters tumor migration, angiogenesis, stromal remodeling, and drug resistance. Monocytes mediate cell death and phagocytosis and direct lymphocyte recruitment, but their effects on stromal remodeling and angiogenesis are influenced by tumor type and tumor localization, and no definitive conclusions can be made [44]. In tumor tissues of patients with breast and gastric cancer, M2-type macrophages are enriched in the tumor microenvironment and their secretions stimulate the migration of tumor cells [45, 46]. The pro- or antitumor effects of mast cells depend on tumor type, stage, and localization. It has been reported that mast cells can cause angiogenesis and lymphangiogenesis in gastric cancer cells and promote epithelial mesenchymal transition [47]. The risk score constructed in this study was positively correlated with the abovementioned immune cells in the tumor microenvironment, suggesting that risk model genes may promote the recruitment of monocytes, M2-type macrophages, and mast cells in the gastric cancer cell microenvironment. T-cell follicular helper plays a key role in tumor adaptive immunity, and its enrichment in tumor microenvironment is related to the prolongation of disease-free survival of patients with liver cancer [48]. CD4^+^ memory T cells were significantly increased in patients treated with antiprogrammed death-1 (PD-1) antibody blockade [49] and their increased abundance in the tumor microenvironment predicted a benign prognosis for patients with gastric cancer [50]. Patients in the high-risk group had lower levels of T-cell follicular helper and memory CD4^+^ memory T cells, suggesting that they may be insensitive to immunotherapy.

The above findings suggest that the present study model can predict the immune status and tumor immune response in gastric cancer patients. Immunotherapy is widely used in inhibiting tumor progression, yet it is not as effective as it could be in stomach cancer patients. MSI is a state in patients with gastric cancer that drives intratumor heterogeneity (ITH) and affects the immune response of the tumor [51]. Among them, where gastric cancer patients with MSI-H are more likely to change to checkpoint inhibitors than MSI-L, we found that patients with high risk scores had a microsatellite status more inclined to MSI-L. Some of the mutated genes in tumors can form neoantigens on tumor cells, and TMB can represent these antigen loads [26]. Studies have shown that high TMB is an effective factor for the application of immune checkpoint inhibitors [52]. We calculated the TMB of each sample based on the mutation data provided by TCGA database; patients in the high-risk group can provide significantly fewer antigen recognition sites than those in the low-risk group. Combined with the MSI results, it is reasonable to conclude that patients in the high-risk group were less effective in receiving immunotherapy.

Although the data of our study came from TCGA and GEO database, the sample size and data of clinical surgical treatment, chemotherapy, and radiotherapy are relatively insufficient and lack of experimental validation *in vivo* and *in vitro*. However, in this study, we created a DDR-related gene model and validated its practical value in predicting the prognosis and immunotherapy efficacy of gastric cancer patients, and we also identified the practical value of DDR genes as a novel therapeutic direction and provided new ideas for clinical practice.

## Figures and Tables

**Table 1:** Patients in public database.

	TCGA (*n* = 337)	GEO (GSE84437, *n* = 433)
Gender		
Female	119	137
Male	218	296
OS		
Alive	197	224
Dead	140	209
T stage		
T1	15	11
T2	74	38
T3	156	92
T4	88	292
Unknown	4	
N stage		
N0	99	80
N1	91	188
N2	68	132
N3	68	33
Unknown	11	
M stage		
M0	303	
M1	22	
Unknown	12	
Stage		
I	45	
II	107	
III	137	
IV	34	
Unknown	14	
Age		
<65	145	267
≥65	189	166
Unknown	3	

## Data Availability

The data that support the finding of this study are openly available in https://portal.gdc.cancer.gov/ and https://www.ncbi.nlm.nih.gov/geo/.

## Supplementary Materials

Supplementary Materials Supplementary Figure 1: in this study, the NMF unsupervised consensus clustering algorithm was used to classify patients into multiple subtypes, and Supplementary Figure 1 shows (*k* = 2‐10) the full subtypes. Genes in group C1 were enriched in pathways such as drug metabolism; genes in group C2 were enriched in pathways such as cell cycle and DNA repair. Supplementary Figure 2: based on the transcriptome data and clinical data of colon cancer patients in the TCGA database, we constructed a model using DNA damage response genes. We used K-M curves (DDR signature) to plot survival differences between the high- and low-risk groups, and ROC curves (DDR signature) to test the accuracy of prediction. We also extracted colon cancer risk genes screened by five other investigators and plotted K-M curves and ROC curves by the same method. When compared together, the model we constructed using DNA damage response genes was more predictive of patient prognosis. Supplementary Figure 3: Figure A, based on the clinical characteristics (gender, grade, stage, and TNM) of colon cancer patients in TCGA database, patients were grouped (patients with unknown clinical characteristics or no data were deleted), and the ability of the model constructed in this study to predict the prognosis of patients in each group was tested. K-M curves showed that the risk model constructed in this study is applicable to patients with different clinical traits. Figure B, prognosis-related DNA damage response genes differentially expressed between the high- and low-risk groups in risk models.
