# Supplementary material for: Models for Predicting Response to Immunotherapy and Prognosis in Patients with Gastric Cancer: DNA Damage Response Genes
Source: Biomed Res Int. 2022 Dec 19;2022:4909544. doi: 10.1155/2022/4909544 (PMC9792237; doi:10.1155/2022/4909544)
Supplement: Supplementary Materials — Supplementary Figure 1: in this study, the NMF unsupervised consensus clustering algorithm was used to classify patients into multiple subtypes, and Supplementary Figure 1 shows (k = 2‐10) the full subtypes. Genes in group C1 were enriched in pathways such as drug metabolism; genes in group C2 were enriched in pathways such as cell cycle and DNA repair. Supplementary Figure 2: based on the transcriptome data and clinical data of colon cancer patients in the TCGA database, we constructed a model using DNA damage response genes. We used K-M curves (DDR signature) to plot survival differences between the high- and low-risk groups, and ROC curves (DDR signature) to test the accuracy of prediction. We also extracted colon cancer risk genes screened by five other investigators and plotted K-M curves and ROC curves by the same method. When compared together, the model we constructed using DNA damage response genes was more predictive of patient prognosis. Supplementary Figure 3: Figure A, based on the clinical characteristics (gender, grade, stage, and TNM) of colon cancer patients in TCGA database, patients were grouped (patients with unknown clinical characteristics or no data were deleted), and the ability of the model constructed in this study to predict the prognosis of patients in each group was tested. K-M curves showed that the risk model constructed in this study is applicable to patients with different clinical traits. Figure B, prognosis-related DNA damage response genes differentially expressed between the high- and low-risk groups in risk models. [file 4909544.f1.docx]

Supplementary figure1: In this study, the NMF unsupervised consensus clustering algorithm was used to classify patients into multiple subtypes, and Supplementary Figure 1 shows (K=2-10) the full subtypes. We found that genes in group C1 were enriched in pathways such as drug metabolism and genes in group C2 were enriched in pathways such as cell cycle and DNA repair by GSEA enrichment analysis of typed differential genes.

Supplementary figure2: Based on the transcriptome data and clinical data of colon cancer patients in the TCGA database, we constructed a model using DNA damage response genes. We used K-M curves (DDR signature) to plot survival differences between high and low risk groups, and ROC curves (DDR signature) to test the accuracy of prediction. We also extracted colon cancer risk genes screened by five other investigators and plotted K-M curves and ROC curves by the same method. When compared together, the model we constructed using DNA damage response genes was more predictive of patient prognosis.

Supplementary figure3：Figure A, Based on the clinical characteristics (Gender, Grade, Stage, TNM) of colon cancer patients in TCGA database, patients were grouped (patients with unknown clinical characteristics or no data were deleted), and the ability of the model constructed in this study to predict the prognosis of patients in each group was tested. K-M curves showed that the risk model constructed in this study is applicable to patients with different clinical traits. Figure B, Prognosis-related DNA damage response genes differentially expressed between high and low risk groups in risk models.


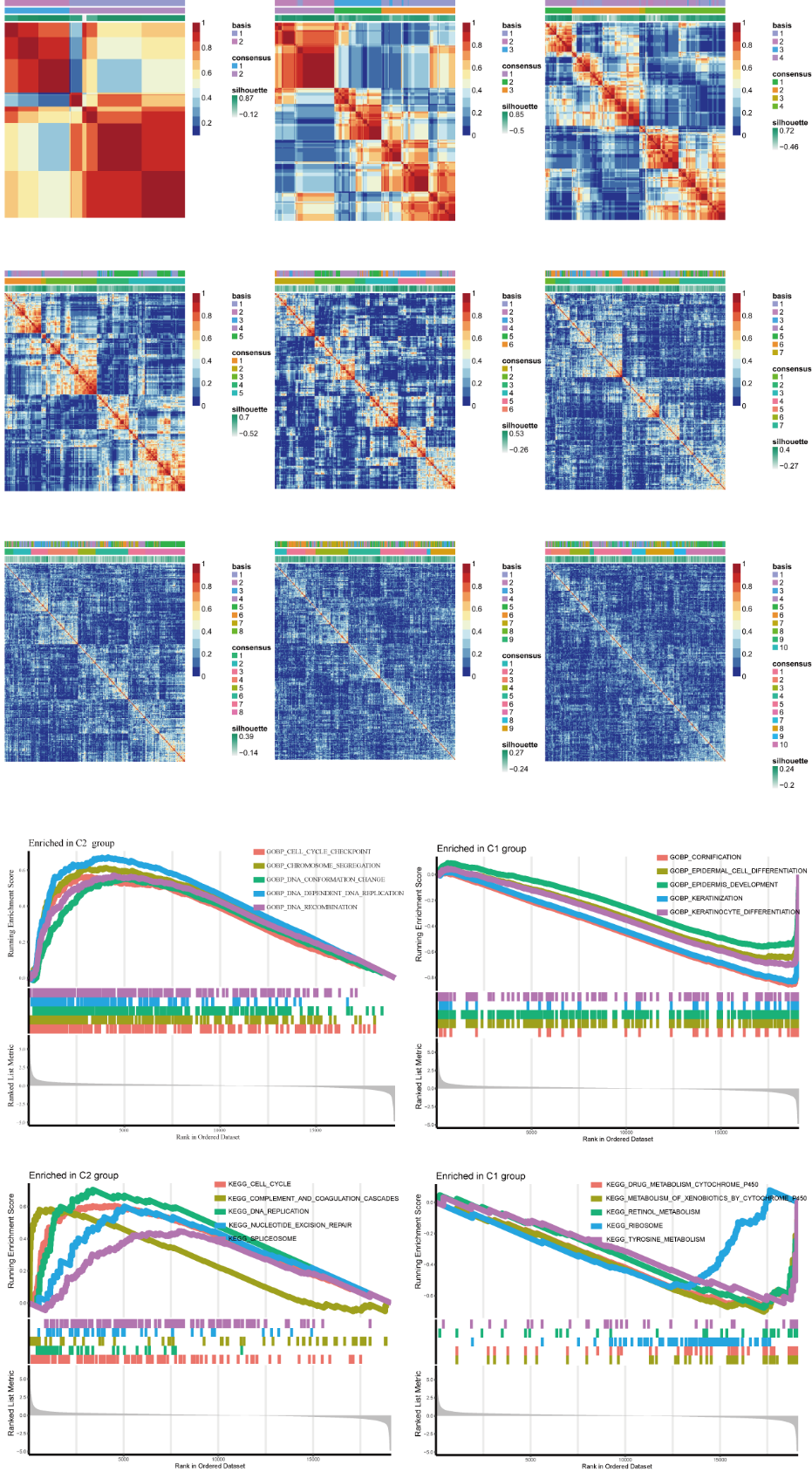


Supplementary figure1：All results of consensus clustering and GSEA.


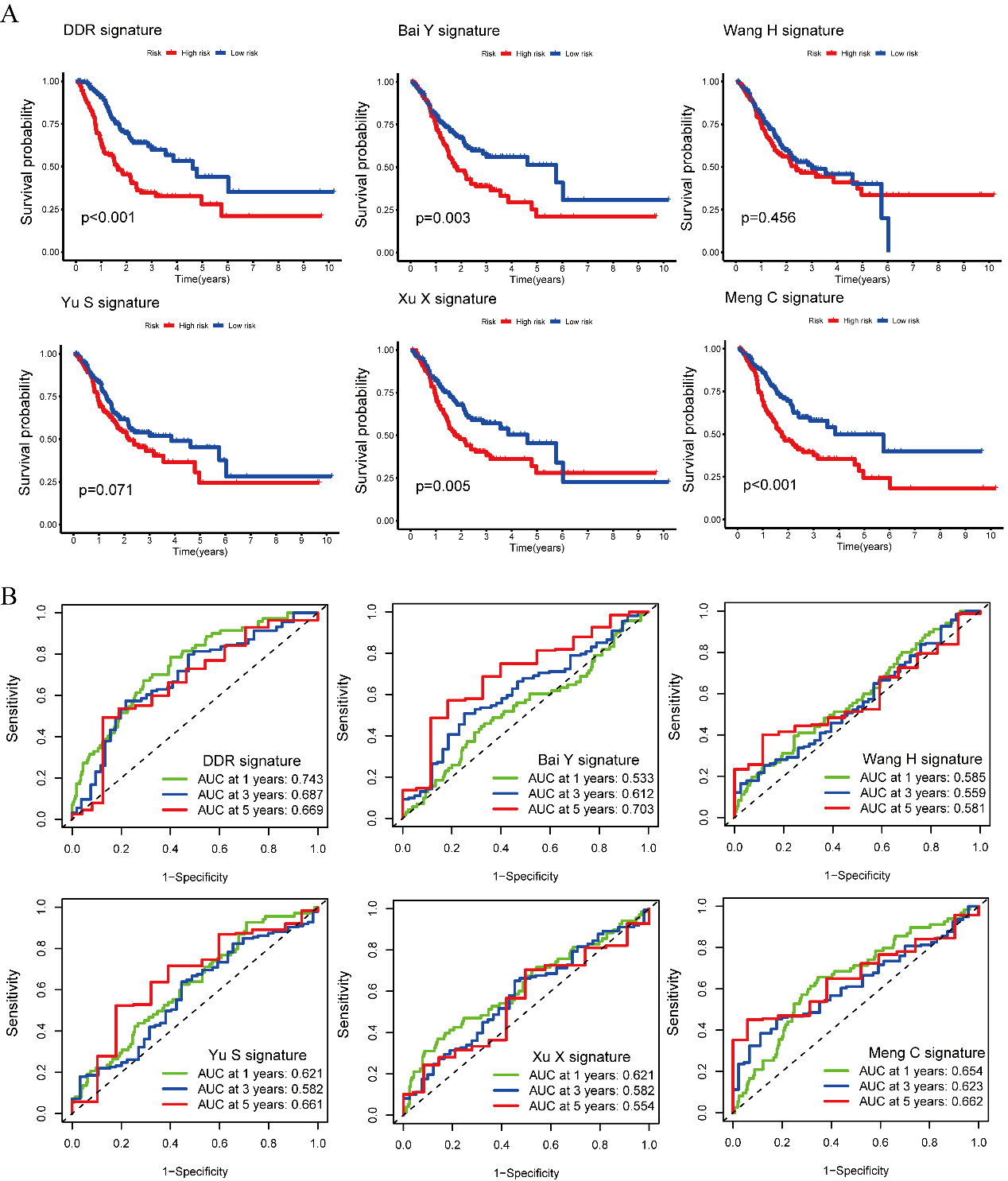


Supplementary figure2：Comparison of prognostic models（A）Kaplan-Meier curves of our prognostic models and others’.（B）ROC curves of our prognostic models and others’.


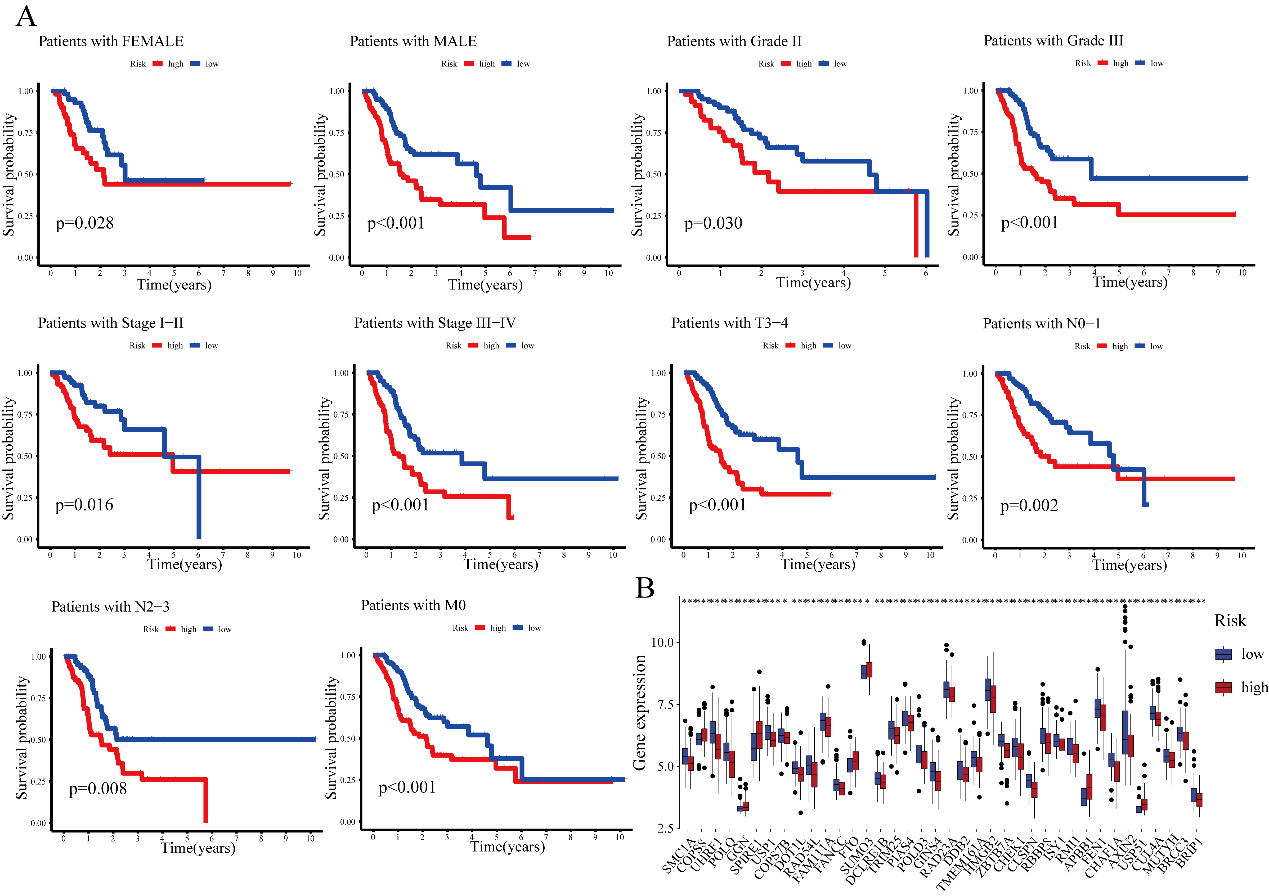


Supplementary figure3：Survival analysis and differential genes（A）Kaplan-Meier curves of patients with different clinical traits.（B）Prognosis related DDR differential genes between high-risk and low-risk groups.
